# Incidence of type 2 diabetes in Aboriginal Australians: an 11-year prospective cohort study

**DOI:** 10.1186/1471-2458-10-487

**Published:** 2010-08-17

**Authors:** Zhiqiang Wang, Wendy E Hoy, Damin Si

**Affiliations:** 1 Centre for Chronic Disease, School of Medicine, University of Queensland, Herston 4029 QLD, Australia

## Abstract

**Background:**

Diabetes is an important contributor to the health inequity between Aboriginal and non-Aboriginal Australians. This study aims to estimate incidence rates of diabetes and to assess its associations with impaired fasting glucose (IFG) and impaired glucose tolerance (IGT) among Aboriginal participants in a remote community.

**Methods:**

Six hundred and eighty six (686) Aboriginal Australians aged 20 to 74 years free from diabetes at baseline were followed for a median of 11 years. During the follow-up period, new diabetes cases were identified through hospital records. Cox proportional hazards models were used to assess relationships of the incidence rates of diabetes with IFG, IGT and body mass index (BMI).

**Results:**

One hundred and twenty four (124) new diabetes cases were diagnosed during the follow up period. Incidence rates increased with increasing age, from 2.2 per 1000 person-years for those younger than 25 years to 39.9 per 1000 person-years for those 45-54 years. By age of 60 years, cumulative incidence rates were 49% for Aboriginal men and 70% for Aboriginal women. The rate ratio for developing diabetes in the presence of either IFG or IGT at baseline was 2.2 (95% CI: 1.5, 3.3), adjusting for age, sex and BMI. Rate ratios for developing diabetes were 2.2 (95% CI: 1.4, 3.5) for people who were overweight and 4.7 (95% CI: 3.0, 7.4) for people who were obese at baseline, with adjustment of age, sex and the presence of IFG/IGT.

**Conclusions:**

Diabetes incidence rates are high in Aboriginal people. The lifetime risk of developing diabetes among Aboriginal men is one in two, and among Aboriginal women is two in three. Baseline IFG, IGT and obesity are important predictors of diabetes.

## Background

Diabetes is an important cause of coronary heart disease [1] and renal failure in Aboriginal people [2], contributing considerably to the 17 year life expectancy gap between Aboriginal and non-Aboriginal Australians. The prevalence of diabetes in Aboriginal Australians is higher than that in the general Australian population [3]. Most of the available data have been from cross-sectional studies. The incidence of diabetes in Aboriginal people from an 8-year follow-up study has been reported [4]. However, there are no data on age-specific and cumulative incidence, which is important information regarding individuals' risks of developing diabetes during a defined period.

Impaired glucose tolerance (IGT) and impaired fasting glucose (IFG) have been defined by the World Health Organisation [5]. The American Diabetes Association (ADA) Expert Committee on the Diagnosis and Classification of Diabetes Mellitus has recommended lowering the diagnostic threshold of IFG from 6.1 to 5.6 mmol/L, which supposedly results in a similar prevalence of IFG and IGT and increases the concordance between the two groups with respect to risk definition [6]. However, people with IFG may have a different background risk when compared with those with IGT[7]. Predictive values of IFG and IGT for risk of incident diabetes in Aboriginal people have not been investigated.

In this study we aimed to i) describe incidence rates of diabetes in an Aboriginal cohort with a median of 11 years of follow-up; and ii) examine relations of incident diabetes with the presence of baseline IFG, IGT and body mass index (BMI).

## Methods

In 1992 a community-wide screening program was initiated in a remote Aboriginal community in Australia's Northern Territory. Participants were offered a baseline examination and testing between 1992 and 1995. Eight hundred and ninety seven (897) adults aged 20 to 74 years (representing over 80% of the adult population in the community) were included in the program. Baseline plasma glucose concentrations were measured in 802 participants: 414 participants had 75 g oral glucose tolerance tests (OGTT); 126 had fasting plasma glucose tests without OGTT; and 262 had random plasma glucose tests only. The remaining 95 participants had no baseline plasma glucose tests and were excluded in this analysis.

Of those 802 participants, 71 had clinically diagnosed diabetes before the baseline examination, and 45 had a new diagnosis of diabetes made at the baseline examination according to World Health Organisation (WHO) 1999 criteria [5]. The remaining 686 participants (321 females and 365 males) free from identifiable diabetes at baseline were followed up to 13 years.

The project was approved by the Behavioural and Social Science Ethical Review Committee of the University of Queensland.

### Measurements

New diabetes cases that occurred during the follow-up period after the baseline examination were identified through reviewing hospital records according to ICD-9 CM code of 250 and ICD-10-AM code of E11, and outpatient clinical records. Only the records related to the first documented diagnosis of diabetes for each individual were used for the analysis. All participants' hospital records were followed up to 30 April 2005. For those who were diagnosed as having diabetes during the follow-up period, their follow-up time was from the time of initial screening visit to the time of having a diagnosis first documented in hospital records. Those who had not reached an endpoint were considered "censored" at the date of 30 April 2005. Participants who had no documented diagnoses of diabetes in hospital records and had died before the end of follow-up were censored at the time of death.

An individual with a baseline fasting plasma glucose concentration of 5.6 - 6.9 mmol/L was considered as having IFG while those with a 2 hour plasma glucose (or random glucose) of 7.8 - 11.0 mmol/L as having IGT. Body weight and height were measured at the baseline examination for calculation of BMI (kg/m^2^). Other baseline measurements included smoking and drinking status, blood pressure, serum cholesterol and urine albumin creatinine ratio (ACR). Detailed baseline data collection methods have been reported elsewhere [3].

### Statistical analysis

The data were partitioned into age bands of < 25, 25-34, 35-44, 45-54 and 55+ years. For individuals who fell into more than two age bands during the follow-period, their total follow-up time was subdivided and allocated into corresponding age bands as described by Clayton and Hills [8]. Incidence rates were estimated for each age group using the numbers of newly diagnosed diabetes cases divided by the person-years of follow-up. Cumulative incidence of diabetes was estimated using the Kaplan-Meier product-limit method. Rate ratios and their 95% confidence intervals were estimated using the Cox proportional hazards model adjusting for potential confounding factors. All analyses were performed using Stata 10.0 [9].

## Results

The baseline characteristics of the study cohort are presented in Table 1. Among 686 adults who were free from diabetes at baseline, 101 had either IGT alone (58 participants) or IFG alone (36 participants) or both (7 participants). During the follow-up period, 124 of those participants were diagnosed as having diabetes. Incidence rates increased with increasing age, from 2.2 per 1000 person-years for those younger than 25 years to 39.9 per 1000 person-years for those 45-54 years (Table 2). However, there was a slight drop of the incidence rate for those aged 55 years or older (30.5/person-years).

**Table 1:** Baseline characteristics of study participants

	Female (n = 321)	Male (n = 365)
Age (± SD), years	35.0 (13.5)	30.8 (10.0)
BMI (± SD), kg/m2	24.1 (6.2)	22.6 (4.4)
Waist circumference (± SD), cm	89.7 (14.8)	85.2 (12.7)
Systolic pressure (± SD), mmHg	117 (19)	125 (16)
Diastolic pressure (± SD), mmHg	71 (13)	77 (13)
Total cholesterol (± SD), mmol/L	4.3 (1.0)	4.8 (1.1)
IFG/IGT	59 (18%)	42 (12%)
BMI < 25 kg/m^2^	189 (60%)	266 (73%)
25 - 29 kg/m^2^	71 (22%)	77 (21%)
≥ 30 kg/m^2^	58 (18%)	22 (6%)

BMI: body mass index

IFG: impaired fasting glucose

IGT: impaired glucose tolerance

**Table 2:** Incidence rates (per 1000 person-years) of diabetes in Aboriginal people

	Incidence rate (95% CI)		
	
Age group Years	Females	Males	Total
< 25	2.6 (0.4, 18.2)	2.0 (0.3, 13.9)	2.2 (0.6, 8.9)
25-34	22.1 (14.4, 33.9)	7.2 (4.0, 13.0)	12.9 (9.1, 18.2)
35-44	26.4 (17.0, 40.9)	23.0 (15.3, 34.7)	24.5 (18.2, 33.0)
45-54	53.2 (35.1, 80.8)	23.6 (11.8, 47.2)	39.9 (27.9, 57.0)
55+	28.2 (15.2, 52.4)	34.5 (16.5, 72.4)	30.5 (19.0, 49.1)

The cumulative incidence rates are shown in Table 3. Females had a higher incidence rate than their male counterparts. It is estimated that, by age of 60 years, 70% of women and 49% of men expect to have a diagnosis of diabetes.

**Table 3:** Estimated cumulative incidence rates (%)

	Cumulative incidence rate (95% CI)	
	
Age point	Females	Males
25	1.4 (0.2, 9.3)	0.9 (0.1, 6.2)
30	10.0 (5.5, 17.8)	3.0 (1.1, 7.8)
35	21.0 (14.4, 30.2)	7.9 (4.5, 13.6)
40	31.1 (23.2, 40.8)	17.6 (12.2, 25.1)
45	39.2 (3.07, 49.1)	27.9 (20.6, 37.0)
50	55.4 (46.1, 65.2)	36.6 (27.7, 47.2)
55	62.8 (53.2, 72.5)	42.4 (31.7, 55.0)
60	69.9 (60.1, 79.1)	48.6 (36.3, 62.5)

The presence of IFG/IGT at baseline and BMI values were important predictors of diabetes. As shown in Table 4, the presence of either form of impaired glucose regulation (IGT or IFG) at baseline increased the risk of developing diabetes in all BMI groups. Similarly, obesity and overweight increased the risk of incident diabetes in both normal glucose and impaired glucose regulation groups with a dose-response relationship.

**Table 4:** Incidence rates (per 1000 person years) of diabetes by baseline plasma glucose and BMI levels.

	Incidence rate (95% CI)
Baseline normal glucose group	
Normal BMI	8.6 (6.2, 12.0)
Overweight	21.2 (14.1, 31.9)
Obesity	53.2 (36.0, 78.7)
Baseline IFG/IGT group	
Normal BMI	24.6 (13.6, 44.4)
Overweight	66.9 (41.0, 109.2)
Obesity	124.7 (73.8, 210.5)

BMI: body mass index

IFG: impaired fasting glucose

IGT: impaired glucose tolerance

Adjusting for age, sex and BMI at baseline, the rate ratio for developing diabetes among participants with either baseline IFG or IGT was 2.2 (95% 1.5, 3.3) (Table 5). The rate ratios for overweight and obesity at baseline were 2.2 (95% CI: 1.4, 3.5) and 4.7 (95% CI: 3.0, 7.4) respectively, with adjustment of age, sex, and the presence of IGT/IFG at baseline. Even though baseline obesity and impaired glucose regulation significantly increased the risk of diabetes, a considerable proportion of diabetes cases (35 persons or 28%) occurred from those with normal baseline BMI (< 25 kg/m^2^) and plasma glucose values.

**Table 5:** Adjusted rate ratios for developing diabetes by impaired glucose regulation and BMI levels at baseline

Group at baseline	Adjusting for	Rate ratio	95% CI	P value
Normal glucose group	Age, sex, BMI	1.0		
IFG/IGT group		2.2	1.5, 3.3	< 0.0001
				
Normal glucose group	Age, sex, BMI, IGT	1.0		
IFG group		1.7	1.0, 3.0	0.02
				
Normal glucose group	Age, sex, BMI, IFG	1.0		
IGT group		1.7	1.0, 2.8	0.04
				
Normal BMI group	Age, sex, IFG, IGT	1.0		
Overweight group		2.2	1.4, 3.5	0.0003
Obese group		4.7	3.0, 7.4	< 0.0001

BMI: body mass index

IFG: impaired fasting glucose

IGT: impaired glucose tolerance

## Discussion

In this study we found that the incidence of diabetes in Aboriginal people was high, comparable to those in other high risk populations such as Pima Indians [10] and people in Mauritius [11]. The observed diabetes incidence rate among participating Aboriginal adults was higher than those reported in the general Australian population [12] and in European [13,14] and American black and white populations [15]. For example, diabetes incidence rates in Aboriginal women were 4-8 times as high as those in the general Australian women for different age groups, while the corresponding values in Aboriginal men were 2-4 times of their general Australian counterparts [12].

Given the life expectancy of around 60 years for Australian Aboriginal peoples, the cumulative incidence of diabetes at age of 60 years calculated in this study provides an approximate estimate of lifetime risk of developing diabetes in this population. That is, the lifetime risk of diabetes among these Aboriginal men is one in two, and among these Aboriginal women is two in three. A previous study reported that lifetime risk of diabetes was 33% for males and 39% for females in the US [16]. The high lifetime probability of diabetes among Aboriginal Australians calls for implementation of effective primary prevention strategies at the population level. It also calls for a more sophisticated understanding of the nature of diabetes susceptibility in this population.

Results from several longitudinal studies have shown that the presence of IGT/IFG is a significant predictor of the development of diabetes [11,17-19]. This study adopted the new American Diabetes Associate threshold for IFG. Studies in other populations have shown that those with new threshold of IFG are at increased risk of diabetes [7,20]. In our study, IFG and IGT identified distinct groups with 36 participants with IFG only and 58 with IGT only. Their contributions to the prediction of incident diabetes are independent of each other, suggesting that the causes and physiological bases of IFG and IGT may be somewhat different. Raised hepatic glucose output and a defect in early insulin secretion are characteristics of IFG, while peripheral insulin resistance is often characteristic of IGT [21]. The presence of either IFG alone or IGT alone doubled the risk of diabetes compared to the normal glucose group. Even after adjusting for the presence of either IFG or IGT, the other remained to be a significant predictor of diabetes. This supports investigation of both IFG and IGT to capture high risk individuals in Aboriginal populations.

Baseline BMI value is an import predictor of diabetes. Overweight individuals have over twofold risk and obese individuals have over fourfold risk of diabetes relative to those with normal baseline BMI values. This finding is consistent with that from a previous study in Aboriginal populations [4]. BMI has been found to be a significant predictor of diabetes in the US general population [22] and in other high risk populations such as South African Indians [23], Pima Indians [24] and Nauruans [25].

The dose-response relationship between BMI and risk of diabetes exists regardless the presence of IFG/IGT. This is comparable with the results from a study of American Indians which have shown that BMI is a significant predictor of diabetes in both normal glucose tolerance group and IGT group [17]. We found the association between IFG/IGT and diabetes was independent of obesity status. The IFG/IGT group had a higher risk of diabetes in all normal weight, overweight and obese groups. Our findings stress the importance of reducing obesity and managing IFG and IGT on diabetes prevention. However, it should be pointed out that a considerable proportion of diabetes cases were from those with a normal baseline BMI (< 25 kg/m^2^) and normoglycemia, suggesting that other factors might have been important for the development of diabetes in this population. We previously reported that albuminuria was an important predictor of diabetes [26]. Hematocrit [27], inflammatory markers [28-31] and gamma-glutamyltransferase [32] are some potential useful predictors. Assessing novel risk factors in this population is currently in progress.

Some limitations of this study should be pointed out. First, the diabetes cases were identified through reviews of hospital and outpatient records. Ninety two percent of the participants had at least one occasion of hospitalisations during the follow up period. We could miss some diagnoses when diabetes was not severe enough for hospital care. This may underestimate the true incidence. Second, we relied on routinely documented diagnosis information in hospital records for identification of diabetes cases during follow-ups, and misdiagnosis might occur at routine practice. Furthermore, the true date of diagnosis of diabetes was probably earlier than that documented in hospital records. Third, there was potential for loss to follow-up of participants' hospital care due to out-migration to other communities. In reality, the hospital in our study setting is the only hospital serving the entire region, so that most people who had out-migrated to communities within several hundred kilometres, the usual pattern, would still have their hospital care recorded in the centralised hospital data system. Permanent out-migration is very uncommon. Fourth, because the sample size was relatively small for old age groups in this study, the incidence estimates may not be precise, as reflected in the wide 95% confidence intervals. Fifth, the data were collected in a remote community, it remains to be verified if the findings apply to other Aboriginal groups. Caution should be exercised in generalising the findings to the broader Aboriginal population in Australia. Sixth, cumulative incidence rates were estimated based on incidence data collected over one decade rather than over life time. If incidence rates change over time, so does an individual's life time risk of diabetes. Finally, the presence of IGT among some participants was determined according to the postprandial glucose values due to the absence of oral glucose tolerance tests. Some IGT individuals might have been misclassified as normal.

## Conclusions

Diabetes incidence rates are high in Aboriginal people. The lifetime risk of developing diabetes among Aboriginal men is one in two, and among Aboriginal women is two in three. IFG and IGT identify people who have an increased risk of diabetes. Baseline IFG, IGT and obesity are important predictors of diabetes.

## Competing interests

The authors declare that they have no competing interests.

## Authors' contributions

ZW conceived the study design, supervised data collection, conducted data analysis and drafted the manuscript. WH participated in study design, interpretation of results and revising the manuscript. DS contributed to data analysis, interpretation of results and drafting the manuscript. All authors read and approved the final manuscript.

## Pre-publication history

The pre-publication history for this paper can be accessed here:

http://www.biomedcentral.com/1471-2458/10/487/prepub
